# Concomitant Immunotherapy and Metastasis-Directed Radiotherapy in Upper Tract Urothelial Carcinoma: A Biomarker-Driven, Original, Case-Based Proof-of-Concept Study

**DOI:** 10.3390/jcm12247761

**Published:** 2023-12-18

**Authors:** Gaetano Pezzicoli, Francesco Salonne, Vittoria Musci, Federica Ciciriello, Stefania Tommasi, Rosanna Lacalamita, Alfredo Zito, Sara Antonia Allegretta, Antonio Giovanni Solimando, Mimma Rizzo

**Affiliations:** 1 Department of Interdisciplinary Medicine, University of Bari “Aldo Moro”, 70124 Bari, Italy; g.pezzicoli@studenti.uniba.it (G.P.); francesco.salonne@uniba.it (F.S.); v.musci4@studenti.uniba.it (V.M.); f.ciciriello3@studenti.uniba.it (F.C.); 2 Molecular Diagnostics and Pharmacogenetics Unit, IRCCS Istituto Tumori “Giovanni Paolo II”, 70124 Bari, Italy; s.tommasi@oncologico.bari.it (S.T.); r.lacalamita@oncologico.bari.it (R.L.); 3 Pathology Department, IRCCS Istituto Tumori “Giovanni Paolo II”, 70124 Bari, Italy; a.zito@oncologico.bari.it; 4 Radiotherapy Center, UPMC Hillman Cancer Center Villa Maria, 83036 Mirabella Eclano, Italy; saraallegretta@cbhspa.it; 5 Unit of Internal Medicine “Guido Baccelli”, Department of Precision and Regenerative Medicine and Ionian Area-(DiMePRe-J), University of Bari Aldo Moro, 70124 Bari, Italy; antonio.solimando@uniba.it; 6 Medical Oncology Unit, Azienda Ospedaliera Universitaria Consorziale—Policlinico di Bari, 70124 Bari, Italy

**Keywords:** upper tract urothelial carcinoma, immune-checkpoint inhibitors, radiotherapy, abscopal effect, genomic profiling

## Abstract

Metastatic upper tract urothelial carcinoma (mUTUC) has a poor prognosis. Immune checkpoint inhibitors (ICIs) have demonstrated efficacy in patients with metastatic urothelial carcinoma. However, data supporting the use of ICIs in patients with mUTUC are limited. A promising synergy between ICI and concomitant radiotherapy (RT) has been reported in patients with mUTUC. Our research involved a case-based investigation and emphasized the successful integration of different specialists’ skills. Observed after partial urethrectomy procedures for muscle-invasive upper tract urothelial carcinoma (UTUC), the radiological detection of lung metastases prompted us to implement cisplatin-based first-line chemotherapy and molecular characterization in the treatment process. We uncovered alterations in the ERBB2 and FGFR3 genes and mismatch repair deficiency at a molecular level. First-line chemotherapy treatment led to a stable disease, and the patient was started on maintenance immunotherapy with Avelumab. Subsequently, an increase in the size of the lung nodules was described, and the patient received radiotherapy for three lung lesions in combination with immunotherapy. After 3 months, a restaging CT scan reported a complete response, which is still ongoing. We discuss the mechanisms driving RT/ICI synergy and the molecular profile of mUTUC as factors that should be considered in therapeutic strategy planning. Molecular insight enhances the originality of our study, providing a nuanced understanding of the genetic landscape of mUTUC and paving the way for targeted therapeutic strategies. The therapeutic armamentarium expansion encourages the design of a multimodal and personalized approach for each mUTUC patient, taking into account tumor heterogeneity and molecular profiling.

## 1. Introduction

In recent years, the incidence of urothelial carcinoma (UC) has been steadily increasing [1], and treatment scenarios have been rapidly evolving [2]. Upper tract urothelial carcinoma (UTUC) accounts for 5–10% of all UC cases, and its current incidence is approximately 1–2 cases for every 100,000 people [3]. UTUC prognosis is usually worse than bladder UC. Unfortunately, one-third of patients with UTUC develop recurrence and metastases, and about 5% have synchronous distant metastases [4]. The SEER database reports a global 5-year cancer-specific survival of 50% for UTUC patients. However, when dividing by stage, the 5-year cancer-specific survival is 79.3% (95%CI 75.6–82.5) for patients diagnosed with localized disease, 55.5% (95%CI 52.7–58.1) for patients diagnosed with locally advanced disease, and 10.9% (95%CI 8.9–13.2) for patients diagnosed with metastatic disease [5]. Moreover, patients with liver metastases or multiple metastatic organ sites have poorer prognoses [6].

In the therapeutic management of UTUC, platinum-based chemotherapy remains the first-choice option in the neoadjuvant, adjuvant, and metastatic settings. For locally advanced UTUC, neoadjuvant platinum-based chemotherapy has led to lower disease recurrence rates, lower mortality rates, and improved overall survival (OS) compared with surgery alone, according to several retrospective reviews [7,8]. In the post-operative setting, adjuvant chemotherapy with a combination of gemcitabine and platinum resulted in better disease-free survival (DFS) [9]. Finally, in the metastatic systemic setting, cisplatinum-based chemotherapy is the first-line standard, with a median OS range of 14.1–15 months [10]. However, data on the treatment for mUTUC is sparse, and recommendations are mainly based on experience, extrapolated from metastatic urothelial bladder cancer.

The breakthrough in mUTUC treatment included in the International Guidelines [11,12,13] is represented by immune checkpoint inhibitor (ICI)-based immunotherapy. In patients with stable disease, partial response, or complete response after 4–6 cycles of platinum-based chemotherapy, the addition of maintenance immunotherapy with an anti-PD-L1 antibody, namely avelumab, in a JAVELIN-100 phase III trial significantly prolonged OS (median OS, 21.4 months in the avelumab arm vs. 14.3 months in the BSC (best supportive care) arm; HR: 0.69; 95% CI: 0.56–0.86; *p* = 0.001). [14,15] Results from the KEYNOTE 045 phase III trial showed an OS benefit of pembrolizumab, a PD-1 inhibitor, as second-line therapy compared to chemotherapy (10.3 months [95% CI 8.0–11.8] vs. 7.4 months [95% CI 6.1–8.3]) [16,17].

In addition to the solid results of ICIs, the most recent clinical trial results allow us to depict a post-ICI setting. Specifically, the antibody–drug conjugate (ADC) Enfortumab Vedotin was proven to be an effective treatment in all patients with mUC in this setting, including UTUC patients [18]. A more personalized approach in the post-ICI setting comes from the Thor phase III trial, which proved the efficacy of an FGFR-inhibitor (FGFRi), erdafitinib, in mUC patients with FGFR2/3 alteration, with one-third of the study population being composed of UTUC patients [19]. The growing body of evidence on ICI and the new reports on ADC and FGFRi finally made it possible to discuss therapeutic sequences in mUTUC. In this scenario, the role of extensive genomic profiling is expanding: given the first evidence of targeted therapy’s efficacy in the Thor trial, many other targets are under evaluation, such as HER-2, the PI3K/Akt/mTOR axis, and DNA reparation systems.

Multimodal treatments represent a valid current strategy in mUC management. For many years, the use of radiotherapy (RT) for mUC was limited to palliation until more recent positive data on ablative radiotherapy were obtained [20].

The efficacy of radiotherapy in combination with ICI treatment has been reported in several retrospective series [21,22].

In this paper, we present the case of a patient with mUTUC treated with concomitant immunotherapy and metastasis-directed radiotherapy, and we discuss the literature describing similar multimodal approaches in mUTUC and their relative outcomes.

## 2. Case-Based Research

We describe the case of a 49-year-old Italian man in good general clinical condition (performance status of 0 according to ECOG (Eastern Cooperative Oncology Group Performance Status Scale)). The only concomitant medical disorder was hypertension, for which he was receiving adequate pharmacological treatment. The oncological family history revealed a breast neoplasm in the mother and a colon neoplasm in the father, who were both alive after local and systemic cancer treatments.

In July 2021, he consulted his general practitioner for right flank pain and hematuria. An abdominal CT scan revealed grade II right hydronephrosis involving the proximal three-quarters of the ipsilateral ureter, with a distal ureteral wall thickening of 4.5 cm. Serum creatinine was elevated (3.88 mg/dL) due to right ureteral obstruction. Urine cytology test results were negative. Endoscopy excluded the presence of disease in the other regions examined, including the bladder.

**Figure 1:**
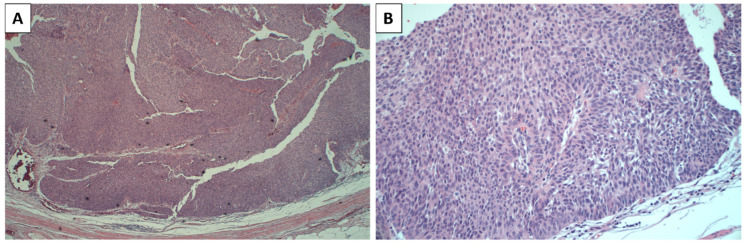
High-grade urothelial carcinoma: the neoplasm completely filled the lumen of the ureter ((**A**)—hematoxylin eosin 4×; (**B**)—hematoxylin eosin 20×).

In September 2021, a partial right ureterectomy and lymphadenectomy were performed, revealing a tumor in the right ureter (extended for 1.4 cm) without lymph node involvement. The pathological diagnosis indicated a high-grade papillary and partly solid urothelial carcinoma (high-grade as per WHO 2016 [23]) infiltrating the muscle layer (pT2 N0) of the ureter (Figure 1).

**Figure 2:**
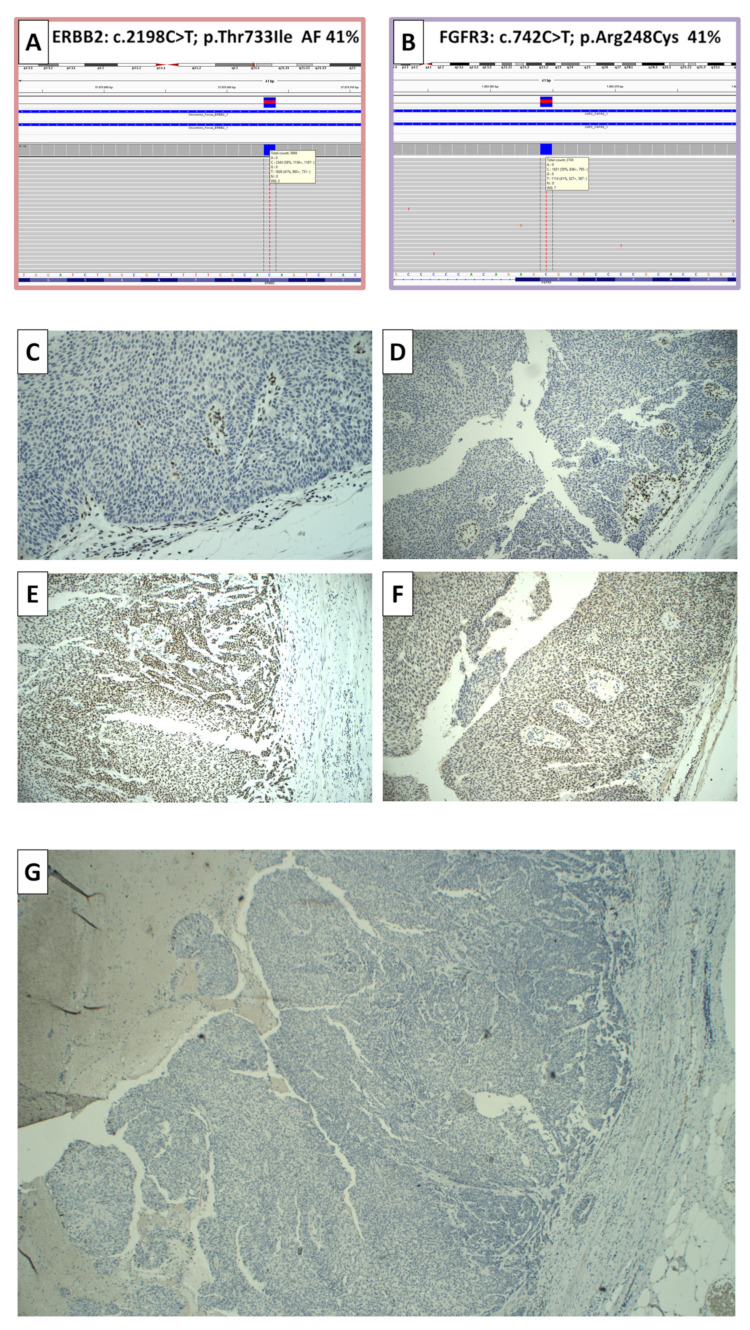
Next-generation sequencing results: ERBB2 mutation (**A**) and FGFR3 mutation (**B**). Immunohistochemical staining of mismatch repair proteins: There was an absence of the MSH2 ((**C**)—20×) and MSH6 ((**D**)—20×) proteins, with internal positive control of lymphocytes. MLH1 ((**E**)—10×) and PMS2 ((**F**)—10×) proteins are normally present. PD-L1 immunostaining (clone 22C3) was completely negative in tumoral cells and in inflammatory cells ((**G**)—4×).

The immunohistochemistry analysis of the mismatch repair proficiency showed the absence of the MSH2 and MSH6 proteins, while the MLH1 and PMS2 proteins were normally present (Figure 2). PD-L1 immunostaining (clone 22C3) was negative in tumoral cells and in inflammatory cells (Figure 2). Based on the immunohistochemical profile, the patient was considered mismatch-repair-deficient (dMMR) and was referred for genetic counseling.

Molecular characterization was performed via NSG analysis on the resected specimen (Oncomine Focus Assay DNA/RNA Thremofisher, Waltham, MA, USA), revealing alterations in the Epidermal Growth Factor Receptor 2 (ERBB2) gene (c.2198C>T; p.Thr733Ile) and the Fibroblast Growth Factor Receptor 3 (FGFR3) gene (c.742C>T; p.Arg248Cys) (Figure 2). The analysis of the ALK, RET, ROS1, FGFR2, NTRK1, NTRK2, and NTRK3 genes did not show signs of alterations. Microsatellite instability (MSI) was also assessed by analyzing eight microsatellite-containing regions (BAT25, BAT26, NR21, NR22, NR24, NR27, CAT25, and MONO27), and the neoplasm was labeled as microsatellite stable (MSS).

Due to the discordance between the evidence of dMMR in IHC and MSS status in NGS, a second extensive NGS genomic profiling process was performed (Oncomine Comprehensive Assay Plus and Oncomine Focus Assay DNA/RNA Thremofisher), revealing the following alterations:

- The MutS Homolog 2 (MSH2) gene (c.1373T>G; p.Leu458Ter; VAF 51% and c.2634+1G>T; VAF 40%) was implicated in DNA repair;
- The lysine N-methyltransferase 2C (KMT2C) gene (c.13913_13914insT; p.Leu4638PhefsTer12; VAF 41%), the 2A (KMT2A) gene (c.3086delA; p.Lys1029ArgfsTer65; VAF 73% and c.3790C>T; p.Arg1264Ter; VAF 70%), the 2D (KMT2D) gene (c.8488C>T; p.Arg2830Ter; VAF 47%), the ATP-dependent chromatin remodeler SMARCA4 gene (c.2653C>T; p.Arg885Cys; VAF 38%), the histone acetyltransferase p300 (EP300) gene (c.4585C>T; p.Arg1529Ter; VAF 40%), and the CREB-binding protein (CREBBP) gene (c.5036CCT; p.Ser1680del; VAF 43%) were all involved in chromatin-remodeling activities;
- The RetinoBlastoma 1 (RB1) gene (c.2117G>A; p.Cys706Tyr; VAF 44%) and the tumoral protein 53 (TP53) gene (c.1010G>A; p.Arg337His; VAF 44%) are both known for their tumor-suppressor roles;
- The ERBB2 mutation (c.2198C>T; p.Thr733Ile; VAF 38%) and FGFR3 mutation (c.742C>T; p.Arg248Cys; VAF 47%) were confirmed.

Moreover, a loss of heterozygosity (LOH) was observed in the region of 11q24.2, comprising the gene CHEK1, which encodes checkpoint kinase 1. Overall, the tumor mutation burden (TMB) was calculated on 1050037 bases and resulted in 26.04 mutations/Mb. Microsatellite instability (MSI) was also assessed, and a high MSI (MSI-H) status was identified.

In November 2021, a CT scan showed pulmonary metastases: 20 nodules in the right lung and 5 nodules in the left lung were observed. The lesions labeled as ‘targets’ according to RECIST 1.1 (Response Evaluation Criteria in Solid Tumors) [24] were one lesion in the upper lobe of the right lung (maximum axial diameter: 13.4 mm) and one lesion in the lower lobe of the right lung (maximum axial diameter: 11.9 mm). Therefore, from November 2021 to March 2022, first-line chemotherapy with gemcitabine and cisplatin (gemcitabine 1000 mg/m^2^, day 1 and day 8, q21d; cisplatin 70 mg/m^2^, day 1, q21d) was administered for a total of six cycles. The patient underwent two re-staging CT scans in January 2022 and March 2022, reporting stable disease according to RECIST 1.1.

Following this, the patient was started on maintenance immunotherapy with avelumab, an anti-PD-L1 antibody, at a dose of 800 mg intravenously every 2 weeks. A subsequent CT scan performed in May 2022, after four immunotherapy administrations, demonstrated an increase in the size of all the known lung nodules (target lesions: upper right lobe, 13.1 mm, and lower right lobe 16.8 mm; the sum of target lesions diameters = 29.9 mm; change from baseline: +18.6%; Stable Disease per RECIST 1.1), but no evidence of new lesions.

The patient underwent a course of volumetric modulated arc radiotherapy, receiving four fractions on two lesions in the lower lobe of the left lung (total dose: 48 Gray) and three fractions on the lesion in the lower lobe of the right lung (total dose: 54 Gray) while continuing the immunotherapy treatment. Specifically, stereotactic body radiotherapy (SBRT) was administered between the fifth and sixth administration of avelumab. In September of 2023, three months after SBRT and after eight more cycles of ICI, a restaging CT scan did not show any of the previous lung lesions, thus configuring a complete radiologic response as per RECIST 1.1. The patient is currently continuing treatment with avelumab, and no recurrences were observed in the most recent chest and abdomen CT scan in September 2023 after the completion of 38 immunotherapy administrations.

**Figure 3:**
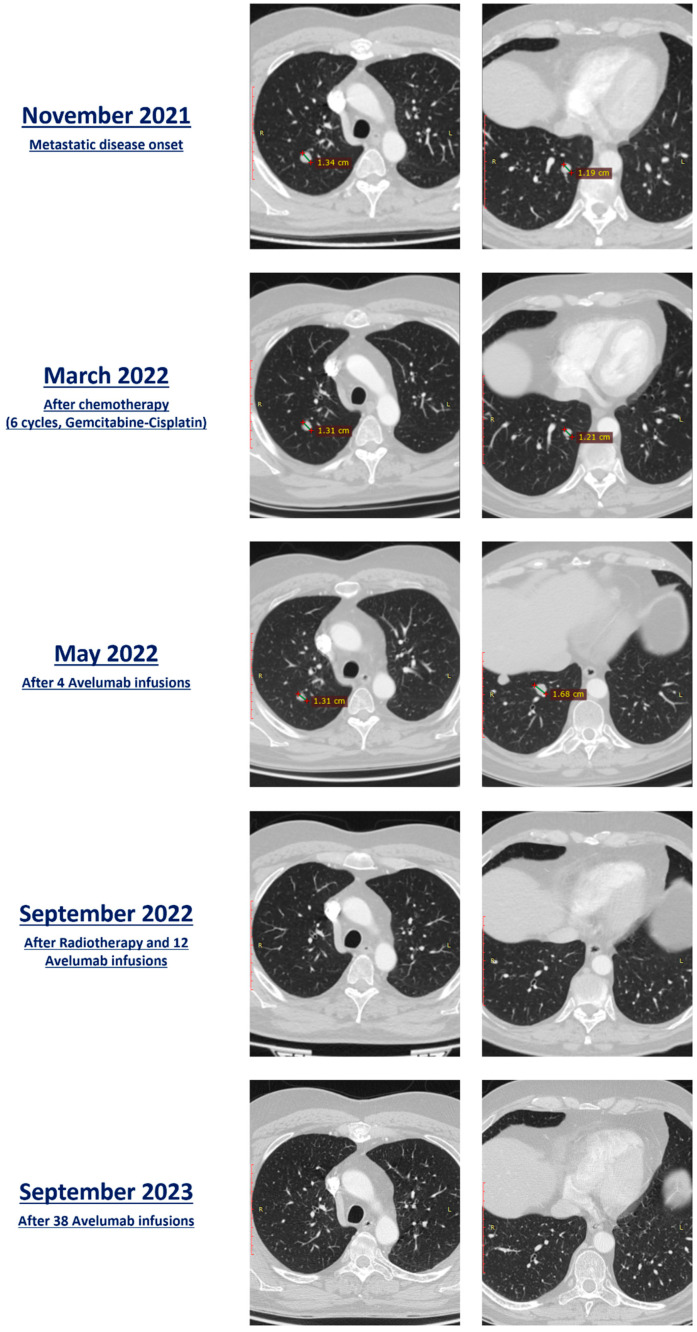
Lung metastasis evolution (target lesions): before the start of therapy (November 2021), after 6 cycles of chemotherapy (March 2022), after 4 administrations of avelumab (May 2022), after radiotherapy and 12 cycles of avelumab (September 2022), and after 38 cycles of avelumab (September 2023).

A detailed summary of the patient’s imaging is given in Figure 3.

The patient did not experience any major adverse events during concomitant SBRT/ICI treatment except for asthenia of grade 1, according to CTCAE (Common Terminology Criteria for Adverse Events) version 5. In the last CT scan, which was performed in September 2023, it was evident that the asymptomatic radiation-induced pulmonary fibrosis (first documented at the September 2022 CT scan) had almost completely disappeared. A urethrocystoscopy performed in October 2023 confirmed the absence of endoscopic recurrence.

## 3. Discussion

### 3.1. Immunotherapy and Radiation Therapy in Urothelial Carcinoma

In our case-based research, the concomitant treatment with ICI during radiotherapy likely contributed to the complete response. Undifferentiated UC is highly radiosensitive [25]. Preclinical studies have documented a synergistic effect between ICI therapy and radiotherapy irradiation which causes the tumor to suffer meaningful oxidative stress, triggering a stress response program that makes tumor cells more immunogenic (by increasing antigen processing and exposure mechanisms). Moreover, oxidative damage can cause changes in protein folding and structure, which could lead to the genesis of neoantigens [26]. Finally, the membrane damage induced by radiation causes cancer cells to rupture, thus spreading antigenic peptides all around [27]. All of these phenomena concur to increase the tumor’s immunogenicity, thus favoring antigen presentation and T-cell priming. The development of specific T-cell responses in the irradiated site could, in some cases, extend beyond the site itself; primed T-cells can reach other tumor sites and exert anti-tumor activity, given that their specific antigen is expressed [28].

The synergy between ICI and radiotherapy has been widely used for the management of solid tumors due to its potential efficacy and optimal safety profile. A pooled analysis of 68 prospective trials showed no significant differences in adverse events between patients treated with ICI monotherapy and patients treated with a combination of ICI and radiotherapy [29]. The efficacy of this combination has also been demonstrated in mUC patients, but mainly in retrospective studies. Traditionally, patients with mUC who received radiotherapy at metastatic sites were treated with palliative intent. However, in more recent reports, the use of ablative radiotherapy has become more frequent. Miranda et al. [20] reported a case series of 52 metastatic bladder cancer patients who received metastasis irradiation, and 40% of them received ablative radiotherapy, reporting 2-year progression-free and overall survival of 19% and 60%, respectively. The ICI plus radiotherapy combination has been described by Sano et al. [21], who retrospectively evaluated the role of concurrent radiotherapy (palliative or ablative) in 24 patients with mUC (4 patients with mUTUC) who received pembrolizumab as a second-line treatment. Patients receiving the combination of ICI and radiotherapy showed longer OS and PFS than patients receiving the ICI alone, even if the data were not statistically significant. Interestingly, patients receiving ablative radiotherapy had a much better OS than patients receiving palliative radiotherapy. Another study from Nakamori et al. [22] retrospectively analyzed a cohort of 235 patients with mUC (92 mUTUC), who progressed after platinum-based therapy and received pembrolizumab as a second-line treatment. The OS was significantly longer in patients who received concomitant palliative radiation (39 patients, 17 mUTUC, median OS: 21 months) compared to patients who received palliative radiotherapy before ICI (32 patients, 12 mUTUC, median OS: 9 months) (*p* = 0.001) and those who did not receive palliative radiation (164 patients, 63 mUTUC, median OS: 13 months) (*p* = 0.019). One of the few prospective works addressing the ICI plus radiotherapy combination is a randomized phase I trial in which 18 pretreated mUC patients received concurrent (9 patients) or sequential (9 patients) pembrolizumab plus stereotactic body radiotherapy (SBRT) [30]. Objective responses were only observed in the concomitant ICI plus SBRT arm (ORR was 0% and 44% in non-irradiated metastatic lesions in the sequential and concomitant SBRT groups). The median OS was 4.5 months for the sequential SBRT group and 12.0 months for the concomitant SBRT group.

The literature data listed above clearly support an additive synergistic effect of the concomitant RT/ICI approach in mUTUC.

### 3.2. Multimodal Treatment and Genomic Profiling in mUTUC

mUTUC is often far more aggressive and refractory to treatments than metastatic bladder cancer. These tumors rarely regress completely after a multimodal treatment approach, as observed in this case. The mechanisms underlying the complete response achieved with concomitant ICI/Radiotherapy treatment in this favorable clinical history remain only partially understood. However, in this paper, we postulate some hypotheses for the therapeutic success which was achieved, including the timing of ICI and SBRT and the predictive value of genetic alterations.

The mechanisms underlying the synergy between radiotherapy and immunotherapy are being increasingly elucidated. Preclinical studies have shown that radiation can enhance antigen presentation, recruit immune cells to the tumor site, and promote immunogenic cell death. Specifically, radiation causes immunogenic oxidative stress and membrane damage in cancer cells, leading to release of neoantigens and tumor antigens. This stimulates dendritic cell activation and cross-presentation of antigens, thereby priming tumor-specific T cells [31]. Additionally, radiation modulates the immune-suppressive tumor microenvironment by diminishing immunosuppressive cytokines and polarizing macrophages towards an immune-stimulating phenotype [32].

This synergy has also been described in our case, in which the avelumab monotherapy received a strong efficacy boost from the radiotherapy. Moreover, a particular outcome reported in our case is tumor regression in unirradiated lesions (22 sub-centimetric lung metastases), known as the abscopal effect, which is achieved with radiotherapy and concomitant ICI. Some cases of the abscopal effect in clinical practice have also been reported in several real-world instances [33,34,35].

Our clinical case demonstrates that carrying out a personalized multidisciplinary therapeutic strategy for each mUTUC patient is crucial. The extent and timing of ICI activity, in particular, is highly variable. Particular attention should be given to the assessment of the response to ICI: while progression to cytotoxic treatment and chemoresistance are well-consolidated concepts, the resistance to ICI treatment has yet to be clearly defined [36]. ICI treatment beyond progression should be considered in selected patients with good performance statuses and persistent clinical benefits during treatment without the appearance of new lesions, according to iRECIST 1.1 criteria [37]. For our patient, maintenance immunotherapy did not initially prove to be effective before radiotherapy triggered immunity. To date, no predictive factors with which to select the best therapeutic approach for an individual mUTUC patient have been validated. Moreover, further prospective studies are needed in order to evaluate the multimodal therapeutic approach in mUTUC patients.

Several clinical trials have combined ICIs with radiotherapy in metastatic urothelial carcinoma patients. Modalities like stereotactic body radiotherapy appear more synergistic than conventionally fractionated radiotherapy, likely due to higher ablative doses per fraction. [38] Ongoing trials like ICONIC (NCT05229614) [39] and CHEERS (NCT03511391) [40] will provide higher-level evidence regarding whether adding radiotherapy to ICIs improves response rates and survival compared to ICIs alone. However, these studies include heterogeneous patient populations and radiotherapy regimens, complicating their interpretation. There is a need for trials optimizing radiotherapy dose-fractionation and evaluating synergy in biomarker-defined subgroups. Another interesting study is the prospective non-interventional study ST-ICI02 (NCT04892849), [41] enrolling patients with solid tumors who receive ICI and radiotherapy as per clinical practice, but they undergo multiple bloods draws to evaluate their immunophenotype and its possible shift during these treatments. This study aims to effectively describe potential predictive biomarkers.

Beyond radiotherapy, other treatment modalities warrant exploration in combination with ICIs for urothelial carcinoma, including immunomodulatory agents, targeted therapies, and chemotherapy. JAVELIN Bladder Medley (NCT05327530) is a phase II, multicenter, randomized, open-label, parallel-arm study that will assess whether the combination of other anti-cancer drugs (Sacituzumab Govitecan, the anti-TIGIT antibody M6223, or the polymer-conjugated human IL-15 called NKTR-255) with avelumab maintenance can contribute to a further improvement in survival for patients with aUC. [42] However, the combination of multiple modalities, also including machine learning, must balance improved efficacy against increased toxicity. Patient quality of life and cost-effectiveness evaluations will be imperative before widely adopting these resource-intensive combination strategies into practice [43].

Another important aspect of our case is the peculiar mutational asset of our patient, who had a mutation of FGFR3 and a mutation of ERBB2. The FGFR3 mutation is known to be frequent in UTUC, more so than in bladder urothelial cancer (35.6% vs. 21.6%) [44]. The impact of FGFR3 mutation on the tumor immune infiltrate has recently been studied with single-cell RNA sequencing in a UTUC tissue specimen: FGFR3-mutated patients had T-cell phenotypes with more active/exhausted Th17-like CD4 cells, lower regulatory T cells, and more CD8/cytotoxic cells in the naïve state, a phenotype that could favor ICI response [45]. Moreover, FGFR3 could be an important actionable oncogene based on the results of the recent Thor phase III trial. [19] The role of ERBB2 mutation in UTUC has been studied more recently. HER2 could be a therapeutic target in mUC; even if an anti-HER2 antibody, trastuzumab, [46] and an anti-HER2 antibody-drug conjugate, T-DM1, [47] were to fail to improve survival in this setting, two ongoing studies, Destiny PanTumor 01 (NCT04639219) [48] and 02 (NCT04482309) [49], are evaluating the efficacy of the promising antibody–drug conjugate trastuzumab deruxtecan in a population that comprises patients with mUC. What makes our patient’s mutational asset unusual is the fact that FGFR3 mutations and ERBB2 mutations are considered mutually exclusive events in UC carcinogenesis [50]. However, this could be explained by the hyper-mutated status of the reported neoplasm: molecular profiling showed a tumor mutational burden of 26 Mutations/Mb, which is quite high, considering that 74% of UC in the TCGA cohort had a TMB lower than 10 mutations/Mb [51]. The MSH2 gene mutations led to the MSI-H/MMRd condition. The potential role of MSI as a predictor of ICI response has also been discussed in mUC: Ma et al. reported a case of an MSI-H patient with mUC that experienced a sustained and prolonged response to ICI (sintilimab, an anti-PD-1 antibody) [52]. Sarfaty et al. reported a retrospective observation of 1333 mUC patients, of whom 26 were MSI-H. In this study, MSI-H was correlated with deep and durable responses to ICI, while it was associated with poor responses to platinum-based chemotherapy [53]. In addition, the extended genomic profiling showed pathogenetic mutations of TP53 and RB1, two tumor suppressor genes. Their mutation was described frequently in high-TMB UC [51].

Another result of the extended genomic profiling is the evidence of multiple mutations in chromatin remodeling genes. The pleiotropic effect of these genes makes it difficult to establish a direct consequence of their mutations. More genomic profiling data could be useful to understand their true role in cancer cell survival and, perhaps, their potential clinical significance.

The validation of molecular biomarkers could be of considerable importance for tailoring immunotherapy, as discussed above. PD-L1 status has limited utility, but it has been proven that patients with PD-L1 positive mUC receiving avelumab had a significantly increased median objective response rate (13.8%), median progression-free survival (5.7 months), and a median 12-month overall survival rate (79.1%) compared to patients receiving the best supportive care (1.2%, 2.1 months, 60.4%, respectively). [54] Another proposed biomarker for immunotherapy is microsatellite instability. In 2017, MSI-H was recognized as the first tissue/site-agnostic indication for the use of the ICI pembrolizumab (an anti-PD1 antibody) based on the result of five single-arm, multicohort, multicenter trials (KEYNOTE-016, -164, -012, -028, and -158). Tumors with MSI-H are well known for their good response to immunotherapy due to their hypermutated status, which leads to a high number of neoantigens [55]. The role of MSI-H as a predictor of the ICI response in mUC has already been described in the previous chapter. This should be taken into consideration when designing a strategy for mUC patients, since MSI-H is a feature present in 6% of the whole population and in even more mUTUC patients (9%) [56].

The patient described herein was dMMR and PD-L1 negative, and experienced a good response to ICI. As discussed above, MMR deficiency could contribute to explaining the depth and duration of response experienced by our patient. On the other hand, PD-L1 negativity did not negatively impact the ICI response. PD-L1 expression is considered a biomarker for ICI response in some tumors, although its intrinsic limitations should be reflected upon; even if PD-L1 is expressed, the lack of adequate immune infiltration into the tumor could significantly limit the efficacy of ICI treatment. Vice versa, the absence of PD-L1 in the tumor tissue does not prevent the systemic activity of ICI, which enhances immune system efficacy.

Therefore, the search for a new biomarker needs to take into account not only the cancer cells’ characteristics, but also how the immune system responds. A possible suggestion comes from ancillary analyses of the ABACUS trial [57], in which the efficacy of atezolizumab, another anti-PD-L1 antibody, was prospectively assessed in patients with muscle-invasive bladder cancer. It was observed that the therapeutic effects of ICI, including survival and the rate of complete responses, were significantly higher in patients with an increased number of CD8-positive T lymphocytes in cancer tissue.

The extensive genomic profiling approach shown in this paper could be extremely useful in detecting potential biomarkers. However, it has some limitations that make its application in common clinical practice difficult: the inaccessibility of genomic profiling and the lack of data validation.

Access to genomic testing in clinical practice is often out-of-scope for smaller hospitals; however, the option of remote testing (such as FoundationOne or Guardant) should be considered. Moreover, the integration of different skills from different professionals could be the winning strategy; the development of molecular tumor boards including molecular pathologists, biologists, bioinformaticians, geneticists, medical oncologists, and pharmacists should be strongly encouraged. Molecular tumor boards could also optimize resources, thus allowing genomic profiling in everyday practice. Moreover, the growing accessibility of genomic profiling will lead to an increase in clinical genomic data pro-duction, which will lead to an easier validation of the integrated models.

Beyond these limitations, molecular profiling of mUTUC is a field undergoing constant innovation, with the introduction of strategies that improve and complete the data derived from tumor tissue DNA analysis. Liquid biopsy, a series of techniques aimed at acquiring information about cancer from its circulating by-products (e.g., circulating tumor cells, circulating tumor DNA (ctDNA), cancer-derived extracellular vesicles) could contribute to biomarker detection. Specifically, it has been demonstrated in mUC patients that ctDNA represents a bona fide representation of tissue tumor DNA [58]. Moreover, since ctDNA comes from all active tumor sites, it could offer a better landscape of tumor heterogeneity, potentially overcoming the intrinsic limit of tissue biopsy. Its predictive role in mUC is supported by the aforementioned phase I trial by Sundhal et al. [28]: a decrease in the ctDNA fraction was associated with the response to concomitant ICI/RT treatment. The periodic, repeated use of a non-invasive procedure such as ctDNA could help to track tumor evolution and anticipate therapeutic adjustments [59].

Finally, an even more accurate molecular biomarker could come from gene expression profiling. It has been demonstrated that characterizing the expression of 150 genes allows for the identification of tumors with pro-immune microenvironments and tumors with non-immune microenvironments, with the first class being much more responsive to ICI [60].

## 4. Conclusions and Future Directions

Our manuscript confirms the relevance of a tailored therapeutic approach to mUTUC. Multimodal treatments should be considered in the management of mUTUC patients receiving ICI, and the therapeutic option that can best synergize with immunotherapy should be preferred. mUTUC molecular characterization targets predictive biomarker validation, thus further customizing the therapeutic approach. Ongoing research will confirm whether liquid biopsy can help to overcome the limitations arising from tumor heterogeneity and recognize individual tumor adaptations to treatments over time. Integrating longitudinal molecular monitoring and multiple treatment modalities in a personalized, adaptive approach based on a patient’s dynamics over time may help to achieve major and durable objective responses and prolong survival. In the coming years, we expect that mUTUC clinical practice will be influenced by the ongoing clinical trial outcomes evaluating the intensification of immunotherapy with metastasis-directed therapeutic approaches or with other drugs synergistic to ICIs.

## Data Availability

The data presented in this study are available on request from the corresponding author.
